# Semi-supervised learning for topographic map analysis over time: a study of bridge segmentation

**DOI:** 10.1038/s41598-022-23364-w

**Published:** 2022-11-08

**Authors:** Cheng-Shih Wong, Hsiung-Ming Liao, Richard Tzong-Han Tsai, Ming-Ching Chang

**Affiliations:** 1 Center for Geographic Information Science, Research Center for Humanities and Social Sciences, Academia Sinica, Taipei, 115201 Taiwan; 2 Computer Science Department, University at Albany, State University of New York, Albany, NY 12222 USA

**Keywords:** Environmental sciences, Environmental impact

## Abstract

Geographical research using historical maps has progressed considerably as the digitalization of topological maps across years provides valuable data and the advancement of AI machine learning models provides powerful analytic tools. Nevertheless, analysis of historical maps based on supervised learning can be limited by the laborious manual map annotations. In this work, we propose a semi-supervised learning method that can *transfer* the annotation of maps across years and allow map comparison and anthropogenic studies across time. Our novel two-stage framework first performs style transfer of topographic map across years and versions, and then supervised learning can be applied on the synthesized maps with annotations. We investigate the proposed semi-supervised training with the style-transferred maps and annotations on four widely-used deep neural networks (DNN), namely *U-Net*, *fully-convolutional network (FCN)*, *DeepLabV3*, and *MobileNetV3*. The best performing network of U-Net achieves $F1_{inst:0.1} = 0.725$ and $F1_{inst:0.01} = 0.743$ trained on style-transfer synthesized maps, which indicates that the proposed framework is capable of detecting target features (bridges) on historical maps without annotations. In a comprehensive comparison, the $F1_{inst:0.1}$ of U-Net trained on *Contrastive Unpaired Translation (CUT)* generated dataset ($0.662 \pm 0.008$) achieves 57.3 % than the comparative score ($0.089 \pm 0.065$) of the least valid configuration (MobileNetV3 trained on *CycleGAN* synthesized dataset). We also discuss the remaining challenges and future research directions.

## Introduction

Large-scale scanning and digitization of historical maps in recent year has provided valuable data that enable automatic machine learning algorithms to be applied. Topographic maps contain rich information of geographic features such as transport networks, population settlement distributions, toponyms, landscape status, etc. These maps are valuable for expediting research on geographic changes for numerous aspects including political, social, or environmental studies. However, human interpretation and annotation of historical maps take considerable time and might require tremendous amount of efforts. The need of automatic map analysis and understanding tools has become an emerging trend. In this work, we develop a semi-supervised learning framework that enables the learning of map analytic models (such as the detection and segmentation of features or legends) to be transferred and applied across versions of maps. This framework can facilitate geographic study across years or different versions and types of map, including historical analysis or political cross-referencing, without the need of data re-annotation or cross-annotation.

Automatic extraction and understanding of geographic features is an active research area. With the raise of AI and deep learning, deep neural networks have been widely applied to many topics of Geographic Information System (GIS), including geospatial or topographic map analysis and historical map study. With the availability of large-scale labeled datasets and powerful deep networks such as the Convolutional Neural Network (CNN), success has been wildly achieved in topics including road type detection^1^, mine or mineral extent identification^2^, and the extraction of archaeological features from historical map series^3^. The ability to automatically identify geographic features from historical maps can benefit research investigation of the anthropogenic modifications over time. For example, Chen et al..^4^ reconstructed Taiwan land cover categories by applying empirical rules to classify attributes of historical maps. They found that the area of urban land is closely related to population expansion.

The training of fully supervised models on large-scale data typically requires large amount of data, where the annotation is laborious and non-scalable. To this end, semi-supervised learning^5–7^, transfer learning^8–11^, and self-supervised learning^12–14^ can mitigate this issue, however with assumptions and limitations. For the annotations in map understanding, the drawing conventions of cartography from different countries and years are usually very different in the colors and symbols. The legends or signs can vary considerably from map to map, even if they represent the same geographical information. Oftentimes, map annotations are not shareable or transferable to another map, even for identical or overlapping geographical locations. This results in insufficient annotations in both quantity and quality, to perform supervised machine learning on the maps. In order to overcome this issue, semi-supervised learning has been applied in a few early works for obtaining ancillary data for recognize geographic symbols in historical maps. Uhl et al.. propose an automated machine learning framework^15–17^ to extract human settlement symbols from contemporary geospatial data. Duan et al..^18,19^ address the misalignment problems of contemporary vector data using deep learning methods. These works rely heavily on the available contemporary vector data, which imposes strong limitation on the year of the historical maps used for feature extraction. For instance, these models might align a non-existent road or fail to identify a human settlement on a historical map, if these features have disappeared in the contemporary vector data. Thus, performance of the learned analytical models is affected by the geographical variations among different years of the historical maps.

**Figure 1:**
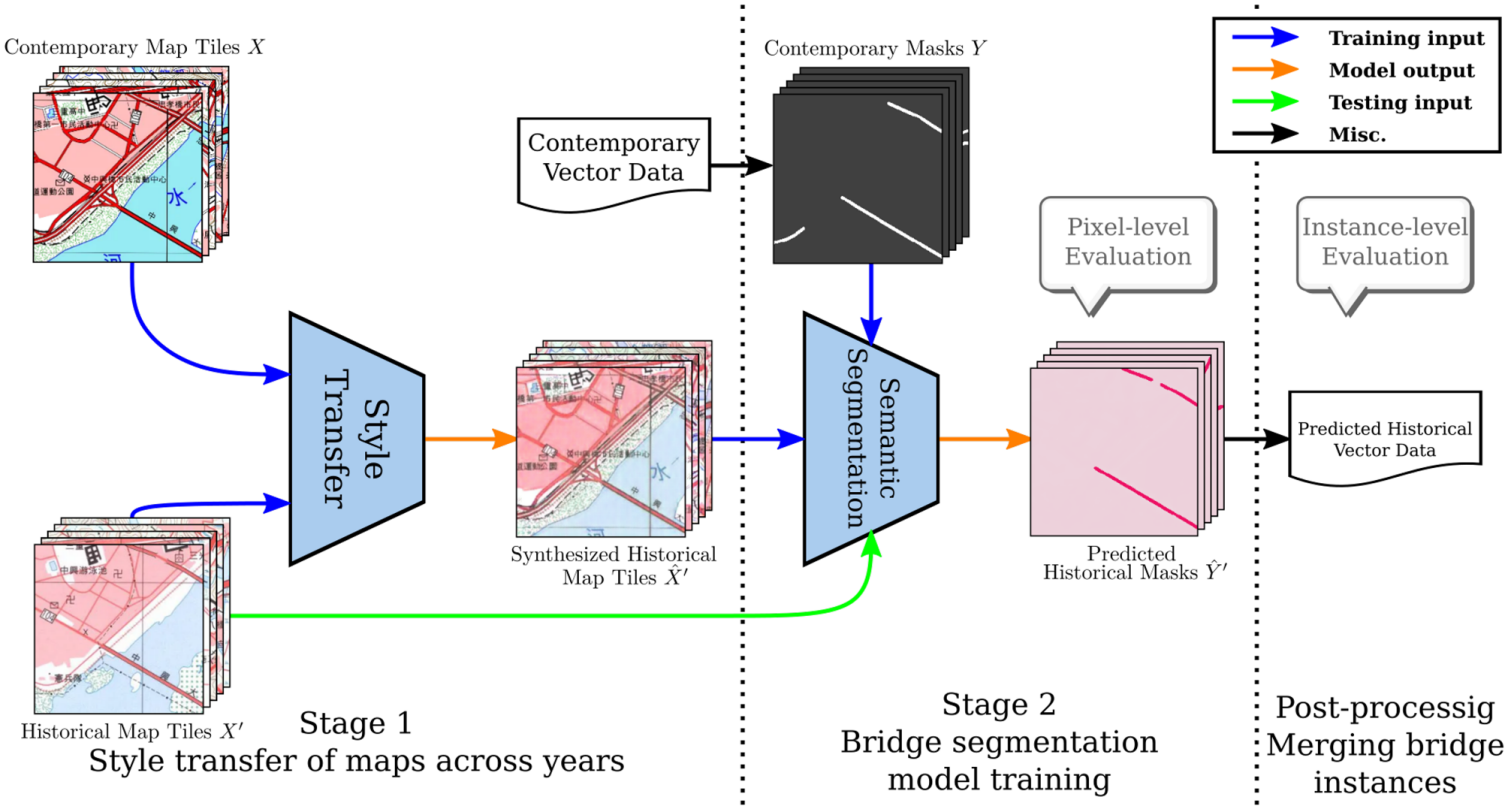
The proposed two-stage framework for cross-year historical map analysis based on semi-supervised learning of map style transfer and bridge segmentation. † The maps in this figure are provided by *Taiwan Historical Maps System* (https://gis.sinica.edu.tw/tileserver/) with permission.

In this paper, we formulate the historical map analysis as a research problem of applying semi-supervised learning to enable the sharing of cross-year annotation data. We assume that geographic information changes gradually over time on the historical map series, and such information can be leveraged via map information fusion. We present a novel **two-stage framework** with focus on the specific problem of bridge segmentation on maps, with the transferring and blending of annotations across historical maps. This way, we can solve the annotation misalignment issues mentioned in the previous paragraph. The first stage of our framework is map image style transfer, which can transfer the content of a map into the style of a desired year that enables map feature sharing across years. The second stage is the training of a deep neural network based on the synthetic map data and augmented annotations that are transferred from other maps. Figure 1 overviews the proposed framework.

Our contributions are summarized as follows:

- *Problem formulation* We formulate the cross-year historical map analysis as a data fusion and semi-supervised machine learning problem.
- *Approach* We propose a novel two-stage framework, where map image style transfer and annotation fusion can effectively deal with the insufficient label issue. We show that model trained this way can detect and segment geographic features across different versions of historical maps.
- *Dataset* We assemble a multi-year historical map dataset consisting of contemporary and historical map tiles, which come with bridge segmentation groundtruth masks covering northern Taiwan.
- *Evaluation* Extensive experiments are conducted for evaluating the performance of three state-of-the-art style transfer networks and four semantic segmentation networks in the proposed framework.

## Methods

### Problem formulation

We formulate the geographic map analysis tasks (e.g. legend recognition or land segmentation) across different versions of maps with partial annotations as a problem of semi-supervised learning for semantic segmentation. The input images consist of map tiles of in all versions or time periods, however the annotations for each version of map might be distinct, lacking in different aspects, or even inconsistent. The problem is then on how best to perform annotation alignment and fusion, such that labels across years can be transferred for used in analysis or model training.

**Figure 2:**
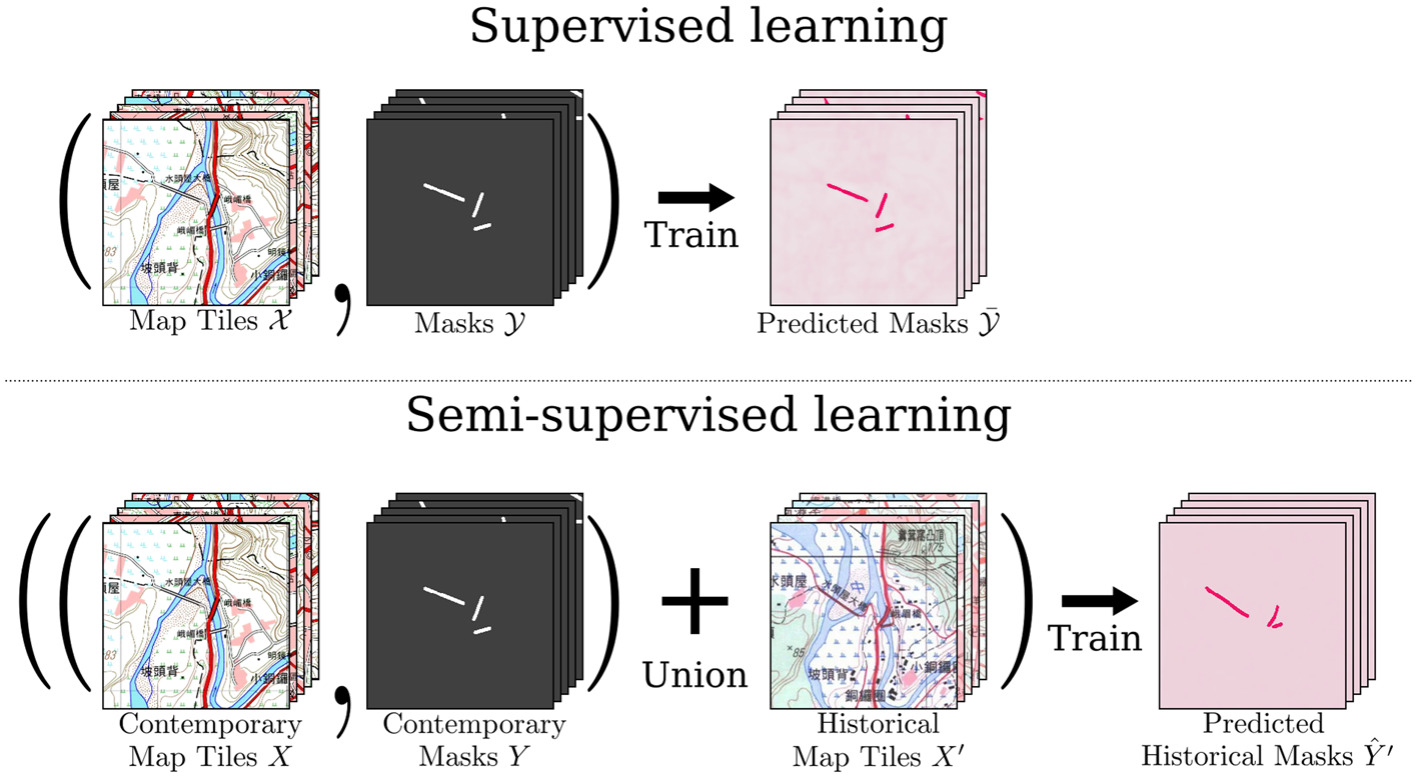
An illustration of supervised vs. semi-supervised learning concepts for applying machine learning for contemporary and historical map analysis. Here the analysis is on automatic bridge detection and segmentation. † The maps in this figure are provided by *Taiwan Historical Maps System* (https://gis.sinica.edu.tw/tileserver/) with permission.

In our semi-supervised setting, groundtruth annotations including the target feature segmentation contours are only available in the contemporary map, while our goal is to train a model that can recognize the target features on maps from different versions or years with distinct styles or legends. Specifically, let $X = \left\{ x_i\right\} _{i=1}^N$ denote the images of the *N* map tiles, and $Y = \left\{ y_i\right\} _{i=1}^N$ denote the groundtruth map annotations of *X*. Let $X' = \left\{ x_i'\right\} _{i=1}^N$ denote the map tiles of the targeted year without annotations that we desire to perform analysis on. Figure 2 shows a conceptual illustration of the proposed semi-supervised learning of models that can analyze geographic features on maps across versions and time.

Our semi-supervised learning framework for enriching and transferring map annotations is based on an assumption, that maps of across multiple versions or years are available, such that the annotations across versions can be transferred for the enrichment of annotations. Our study on the georeferencing of maps across versions or time can bring valuable research insight. For example, the study of a city or country across years can provide valuable information for understanding the chronological development at a larger scale. The analysis across versions of maps provides a way to effectively study the geographical (spatial) and chronological (temporal) relationships.

We hypothesize that data-driven models can be trained using only annotations of a specific map versions, and that such annotations are transferable for model training on other versions of maps. Specifically, our goal is to find a function ${{\mathscr {M}}}$ using the contemporary map tiles *X* with annotations *Y*, together with the historical map tiles $X'$, and predict $Y'$ the geographic features on the historical map tiles, ${{\mathscr {M}}}: \left\{ X, Y, X' \right\} \mapsto Y'$.

In this study, we focus on the analytic segmentation of bridges from maps. We choose the bridges as the targeted feature to extract, as the bridges serve as a crucial role in the basic infrastructure and urban development. Bridges are essential constructions crossing diverse geographic barriers in many countries, especially in Taiwan. There were more than 30,000 bridges^20^ recorded in Taiwan in 2015. Identifying the locations and connectivity of bridges from historical maps can greatly assist the geographical researchers in the investigation of the functionality and migration of bridges over time. The proposed method provides an effective approach to train a bridge segmentation model that can identify and localize bridges on the historical maps, where the annotations of are not available and not required.

### The two-stage framework for bridge segmentation

The proposed framework for cross-year map geographic feature segmentation consists of two stages. The first stage aims to synthesize maps with the style of the historical map, using the contents from the contemporary map. Since the geographic features of the synthesized historical map are transferred from the contemporary map, we expect that the contemporary vector data probably fits the features of the synthesized map. In the second stage, semantic segmentation models can be trained using these image pairs with partial supervision to analyze geographic features on historical maps.

**Figure 3:**
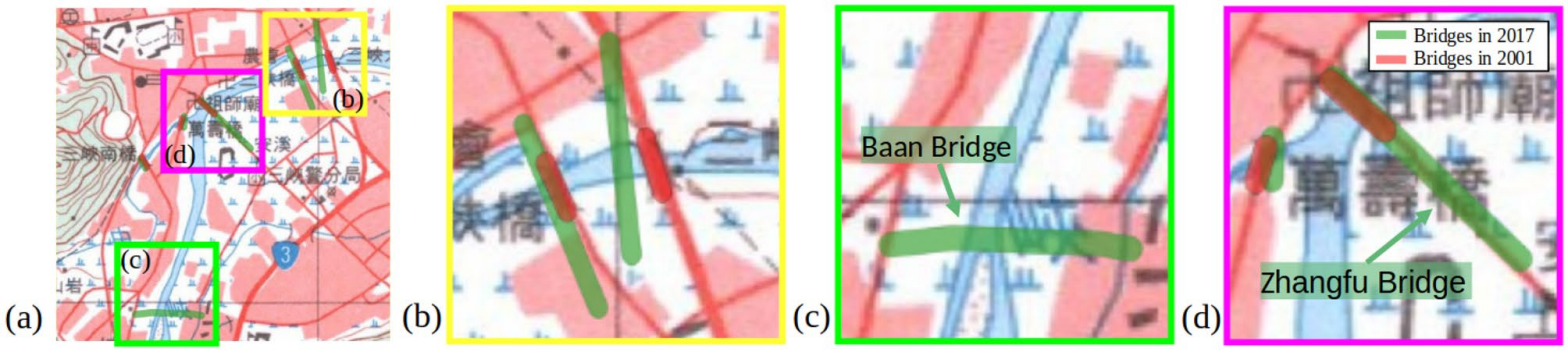
An example of misalignment of geographic features between a contemporary map and a historical map. In (**a**), an example map tile published in 2001 shows some possible misalignment between bridges in contemporary vector data (green strokes) and on historical maps (red strokes), and (**b**), (**c**), and (**d**) are subregions in (**a**) to emphasize the misalignments. In (**b**) bridges had changed greatly by 2001, while in (**c**) the Baan Bridge wasn’t opened until 2007, and Zhangfu Bridge shrank in 2001. † The maps in this figure are provided by *Taiwan Historical Maps System* (https://gis.sinica.edu.tw/tileserver/) with permission.

*Stage 1: Map content transfer and synthesis across versions and years* In the presence of the misalignment issue shown in Fig. 3, we resolve this issue by transferring the content of contemporary maps across versions and years. Since the contemporary vector *Y* does not align with the historical maps $X'$, a style transfer model, Contrastive Unpaired Translation (CUT)^21^, was chosen to synthesize historical maps with contemporary content.

The CUT is a Generative Adversarial Nets (GAN)^22^ based image-to-image translation model that learns a cross-domain similarity function by maximizing mutual information^23^. The GAN model consists of a generator and a discriminator. In our case, the generator *G* generates a historical-look map tile from a contemporary map tile. On the other hand, the discriminator *D* outputs a single scalar of a given map tile *x*, representing the probability that *x* is come from historical maps. The loss function of CUT includes an adversarial loss and a *PatchNCE* loss from the CUT paper. For the adversarial loss, the generator is trying to fool the discriminator by producing map tiles looked historically, but at the same time the discriminator distinguishes between real and fake historical map tiles. The PatchNCE loss maximizes the mutual information between the input contemporary map tiles and the output synthesized historical map tiles.

With this CUT model, map tiles with contemporary content and historical style are synthesized by an optimal generator $G^*$: ${{\hat{X}}}' = G^*(X)$. Thus, the synthesized historical map ${{\hat{X}}}'$ are used to train semantic segmentation model with contemporary groundtruth masks *Y* in the Stage 2.

*Stage 2: The training of the bridge segmentation network* An U-Net^24^ was employed to segment the target features in the proposed framework. The encoder of the U-Net consists five layers downsampling the input map tiles to 64, 128, 256, 512, 1024 channels with two $3 \times 3$ convolutions and one $2 \times 2$ max pooling each layer. Next, the decoder also consists five corresponding upsampling layers, and each upsampling layer does $2 \times 2$ up-convolution halving the number of channels from the previous layer and concatenates features from the corresponding downsampling layers as known as *skip connections*. The output is computed by the final layer of the decoder with a $1 \times 1$ convolution as the number of classes channels: ${\hat{Y}}' = UNet \left( {\hat{X}}' \right)$. We selected the Lovász-Softmax loss^25^ to calculate the segmentation loss, which improves the accuracy of semantic segmentation. The loss is measured on the output of the U-Net ${\hat{Y}}'$ and the contemporary annotations *Y*, and the U-Net is optimized by minimizing the loss with *Adam*^26^ optimizer. That is to say, our model is trained using annotations from a version (2017), and when tested, it is expected to work on another version (2001).

*Post-processing* After masks for the target geographic features are predicted by our model, additional post-progressing steps transform the masks of map tiles into vector data across all map tiles. First, morphological opening operation with ellipse kernel (kernel size 5 pixels) eliminates noisy small pixel groups. Second, morphological closing operation with the same setting of the opening operation is used to complete the shape of target features. The contours of the predicted masks are extracted as vectorized polygons, and final vector data of the features is the union of such polygons across all map tiles.

## Experimental results

We start this section by explaining how we collect the topographic maps and the processing of them. We next describe the evaluation metrics for comparing the performance of bridge segmentation, and present experimental results. We will show that the proposed framework is capable of training a bridge segmentation network to segment bridges on the 2001 historical map, while only uses the contemporary 2017 annotations for training. We will also compare across multiple popular network backbones that are used for semantic segmentation.

### Data source

**Figure 4:**
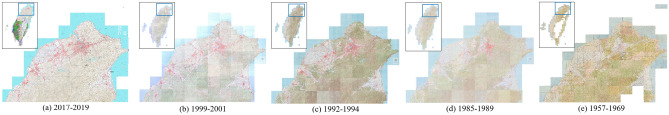
1:25,000 Topographic maps of the northern Taiwan in 2017-2019, 1999-2001, 1992-1994, 1985-1989, and 1957-1969 from the GIS Center of Academia Sinica. The symbol styles of land use such as paddy fields, orchards, grasslands and unused land have been adjusted due to cartographic regulations changes between releases. The same legend was used in the late 20th century (versions b to d) probably for easing the translations between these maps. Refer to the digital version of this paper for detailed zoom-in with full resolution. † The maps in this figure are provided by *Taiwan Historical Maps System* (https://gis.sinica.edu.tw/tileserver/) with permission.

We perform model training and evaluation experiments on the Taiwan historical maps from 1957 to 2017 provided by the *Center for Geographic Information Science (GIS)*, *Research Center for Humanities and Social Sciences* (RCHSS) at *Academia Sinica* (http://gis.rchss.sinica.edu.tw). These maps available through the *Web Map Tile Service (WMTS)* (https://gis.sinica.edu.tw/tileserver), a standard protocol published by the *Open Geospatial Consortium*. Since the 2017-2019 maps tiles of southern Taiwan is still under construction, we conduct experiments on the northern portion of historical maps. Figure 4 shows examples the map tiles in scale of 1:25,000 from 2017-2019, 1999-2001, 1992-1994, 1985-1989, and 1957-1969. Observe the difference of legend used in the maps across years, for example, the thickness and color of line symbols for different types of roads, and the polygon symbols for different land use. The most obvious legend change is the hash pattern of the undeveloped areas including the paddy fields, orchards, grasslands and the unused lands.

In this work, we collected two sets of map tiles from the WMTS server as the training images: (1) the contemporary set published in 2017-2019, hereinafter referred to as 2017 version, and (2) the historical set published in 1999-2001, hereinafter referred to as 2001 version.

We extract the geographic feature annotations of the maps from the *open vector data* provided by the *the National Land Surveying and Mapping Center (NLSC)* of Taiwan (https://maps.nlsc.gov.tw). The open vector data presents present day (2022) geographic features, including roads, farms, temples, lakes, etc., with points, lines, or polygons.

### Data preprocessing

**Figure 5:**
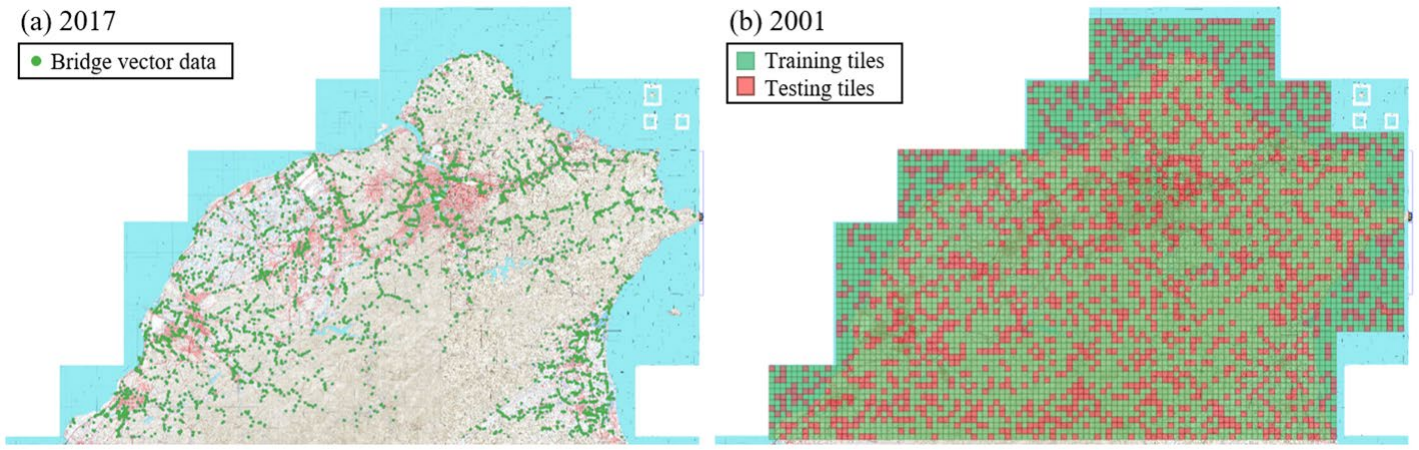
(**a**) Bridge vector data on the 2017 contemporary map of northern Taiwan. Green dots show the 3, 542 annotated bridges from the vector data. (**b**) Train/test split of the 5,986 map tiles at zoom-level 15.Green squares depicts the training tiles and red squares depicts testing tiles. † The maps in this figure are provided by *Taiwan Historical Maps System* (https://gis.sinica.edu.tw/tileserver/) with permission.

For each map version, we manually select 5,986 map tiles at *zoom level 15* and randomly separate the tiles into the training and test sets. The ratio of training vs. test data size is 70 : 30. Next, we use the RoboSat^27^ open-source tool to download the map tiles from the WMTS server at zoom level 15 as $256 \times 256$ images. This way, we create the *contemporary* (${\mathbf {X}}$) and *historical* ($\mathbf {X'}$) map tiles. Figure 5 shows the selected map tiles with bridge annotations and the training/test splitting distribution. Note that some map tiles covering the oceans are selected in our experiments for training and testing. We understand there should not be any bridge in the ocean; however the experiment can verify the false positive of bridge detectors, and we indeed observed zero false-positives of bridge segmentation in our experiment.

**Figure 6:**
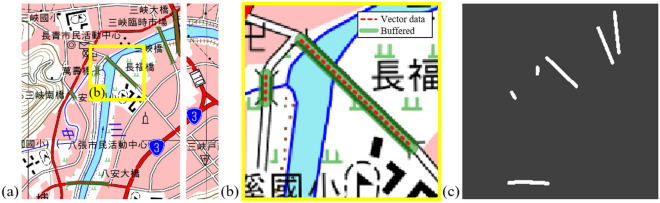
Converting the NLSC open vector data of bridge annotations into masks of the corresponding map tiles. (**a**) shows an example map tile of the labeling from the *buffer operation*, which generates buffers around points, lines, and polygons within a given distance. (**b**) shows a close-up of (**a**), where the vector data of bridges is drawn in red dotted lines, and the corresponding buffering regions are drawn in green strokes. (**c**) shows the extracted bridge masks of (**a**). † The maps in this figure are provided by *Taiwan Historical Maps System* (https://gis.sinica.edu.tw/tileserver/) with permission.

We next describe the steps for collecting groundtruth bridge masks from the NLSC open vector data, in order to train of a machine learning model for bridge segmentation. The bridge annotations are represented as line segments of the bridge starting and ending coordinates in the NLSC vector data. We use the software QGIS^28^ to perform the *buffer operations* on the vector data in transforming the line segments into polygons. The buffer operations is performed with parameter of 0.0001 degree, which is determined empirically such that the masks are large enough to cover the bridges at the given scale. We next rasterize the bridge polygons and turn them into masks on each map tile image. Figure 6 shows an example result of this bridge mask generation. The resulting bridge masks for the corresponding map tiles are ready to used for training the semantic segmentation neural network. To evaluate the effectiveness of the proposed semi-supervised learning framework, we further annotate 1, 210 bridge masks for the historical map tiles as the groundtruth for the test set.

We will release this dataset to the public for reproducibility and to facilitate further research of map analysis.

### Evaluation metrics

**Figure 7:**
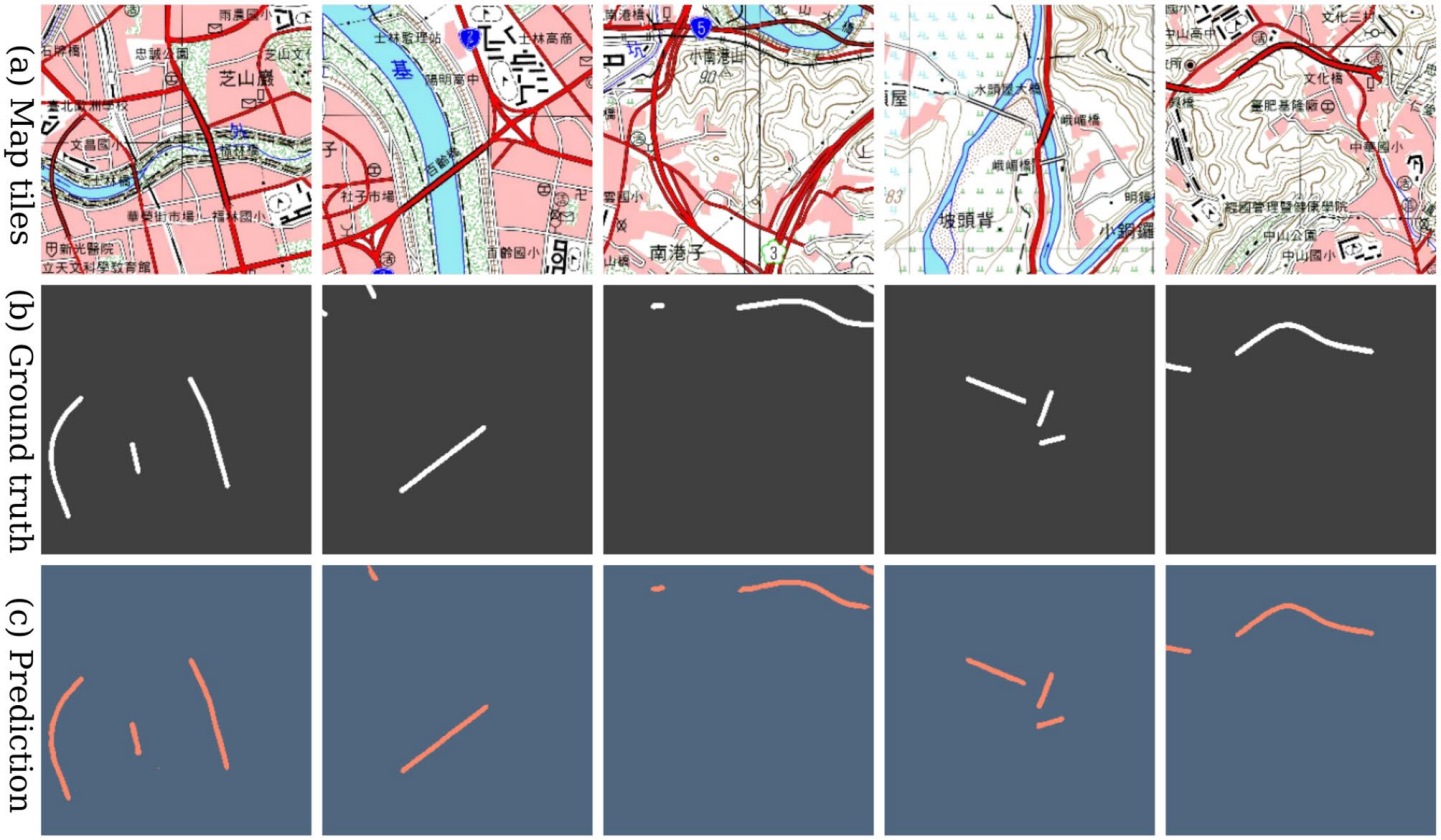
The results made by the U-Net model trained with fully labeled data of the contemporary dataset. (**a**) The contemporary map tiles, (**b**) The groundtruth of bridges symbols, and (**c**) The predictions of bridges on the map tiles. The model predicts almost every bridge symbol on most map tiles successfully. † The maps in this figure are provided by *Taiwan Historical Maps System* (https://gis.sinica.edu.tw/tileserver/) with permission.

To evaluate the per-pixel segmentation performance, we adopt the widely-used *Intersection-of-Union (IoU) Precision-Recall (PR)* as the metrics for semantic segmentation as binary classification in computer vision. The IoU-PR metric can be measured at both the per-pixel and object instance levels.

*Map tile pixel-level PR* Given the per-pixel bridge annotation groundtruth, the pixel-level precision ($P_{px}$), recall ($R_{px}$), $\text {F}_1$-score ($F1_{px}$), and intersection-over-union ($IoU_{px}$) can be calculated between the predicted bridge masks and the groundtruth masks for each map tile or for all map tiles. Scores are obtained by classifying all pixels of 1,796 map tiles of testing set into different error types. Figure 7 shows a visual example. A correctly predicted bridge pixel represents true positive ($TP_{px}$); On the contrary, if a bridge pixel is predicted as background, it is counted as false negative ($FN_{px}$). On the other hand, if a background pixel is predicted as a bridge pixel, the case is a false positive ($FP_{px}$). Finally, the precision-recall analysis does not consider the true negative ($TN_{px}$), which occurs very frequently that a background pixel is predicted correctly as background. Accordingly, the pixel-level PR metrics are defined as:

1 $$\begin{aligned}&P_{px} = \frac{TP_{px}}{TP_{px} + FP_{px}}, \; R_{px} = \frac{TP_{px}}{TP_{px} + FN_{px}}, \;\nonumber \\&F1_{px} = \frac{2 \times P_{px} \times R_{px}}{P_{px} + R_{px}}, \; IoU_{px} = \frac{TP_{px}}{TP_{px} + FP_{px} + FN_{px}}. \end{aligned}$$

*Bridge instance-level PR* The precision-recall can also be calculated to reflect the performance of how well each bridge instance is segmented, when compared with the groundtruth. Here the calculation must be performed after map tiles are combined and tiled back, so the complete mask of each individual bridge can be obtained. The instance-level precision $P_{inst:thres}$, recall $R_{inst:thres}$, and $\text {F}_1$-score $F1_{inst:thres}$ can then be calculated for each bridge. For the next couple lines, we define that if a bridge *intersects* another bridge, the IoU of these two bridges should be greater than a given *threshold*. If a contour of a predicted bridge intersects with a bridge’s contour in the labeled vector data, the contour is regarded as at true positive ($TP_{inst:thres}$). Once again, if it does not intersect with any bridge in the vector data, it is considered as a false positives ($FP_{inst:thres}$). If none of the predicted bridges intersects a bridge contour in the labeled data, the bridge contour is considered as a false negative ($FN_{inst:thres}$). Specifically, the metrics are defined as:

2 $$\begin{aligned}&P_{inst:thres} = \frac{TP_{inst:thres}}{TP_{inst:thres} + FP_{inst:thres}}, \;\nonumber \\&R_{inst:thres} = \frac{TP_{inst:thres}}{TP_{inst:thres} + FN_{inst:thres}}, \;\nonumber \\&F1_{inst:thres} = \frac{2 \times P_{inst:thres} \times R_{inst:thres}}{P_{inst:thres} + R_{inst:thres}}. \end{aligned}$$

Finally, the mean of the instance-level precision, recall, F1-scores then are calculated for all bridges in the test map tiles with IoU threshold is 0.1 (denoted as $F1_{inst:0.1}$). For the purpose of assisting geographers locating potential target features, F1-scores with a lower IoU threshold 0.01 ($F1_{inst:0.01}$) are also provided.

### Results of supervised training of bridge segmentation on contemporary maps

**Table 1:** Quantitative evaluation of the U-Net model trained on the 2017 contemporary map with full supervision. CM Training and CM Testing are the train/test split of the northern Taiwan tiles from 2017 contemporary map. CM Taichung is a separate test set from middle Taiwan of the 2017 contemporary map.

Trained on	Tested on	$P_{px}$	$R_{px}$	$F1_{px}$	$IoU_{px}$	$P_{inst:0.1}$	$R_{inst:0.1}$	$F1_{inst:0.1}$	$F1_{inst:0.01}$	#Bridges
CM Training	CM Testing	0.845	0.764	0.803	0.670	0.935	0.820	0.874	0.893	960
	CM-Taichung	0.986	0.373	0.541	0.371	0.966	0.898	0.931	0.942	1783
	HM Testing	0.849	0.003	0.007	0.003	0.000	0.000	0.000	0.005	3

* CM stands for contemporary map; HM stands for historical map. #Bridges shows the number of recognized bridges.

We evaluate the performance of the method from previous works^2,17^ on segmentation task for geographic feature extraction on historical maps. We will show that semantic segmentation models are effective in detecting and segmenting out bridges from topographic maps with a fully labeled dataset. We trained a U-Net with map tiles $X_{train}$ and masks $Y_{train}$ of the training set on the contemporary map and tested it with the testing set $Y_{test}$. We obtained $F1_{inst:0.1} = 0.874$. Figure 7 show visual results of such bridge segmentation prediction. All experiments are conduct on a NVIDIA GeForce GTX 1080 Ti (11GB Memory per GPU). For U-Net, it takes about 26.9 ms (milliseconds) to perform training on an image and 13.2 ms for running the test on an image.

*Test on unseen maps of another region with the same style/year* To test the generalizability of the bridge segmentation model across maps, we test the trained U-Net on an unseen portion of the contemporary map, namely a region around the Taichung City of middle Taiwan, which is outside the original training and test set of northern Taiwan. Results in Table 1 suggest that the U-Net is generalizable to extract geographic features to unseen maps with the same style/year following this supervised training approach.

*Test on maps with different style/year* We expect that such supervised learning model trained on a contemporary map should not work well across maps of different styles or years. Results from the last row of Table 1 demonstrates this point, where the U-Net trained with the contemporary map *X* can only identify 3 bridges (out of 3,542) in the 2001 historical map $X'$. In other words, the semantic segmentation U-Net cannot learn to recognize geographic features across different map styles effectively, unless it is trained explicitly using maps with those styles.

### Evaluation of semi-supervised training of bridge segmentation on historical maps

This experiment validates how well a model can be trained to recognize geographic features from using solely the style-transferred maps and transferred annotations. We evaluate the proposed two-stage semi-supervised framework using the 2017 contemporary map *X* and 2001 historical map $X'$. We train three style transfer models, which are Pix2Pix^29^, CycleGAN^30^, and Contrastive Unpaired Translation (CUT)^21^, to learn the mapping between *X* and $X'$. The style transfer models are then used to generate a synthesized historical map ${\hat{X}}'$ from *X*. With the synthetic map ${\hat{X}}'$ and the contemporary masks *Y* as annotations, four semantic segmentation models are trained in Stage 2: U-Net^24^, fully-convolutional network (FCN)^31^, DeepLabV3^32^, and MobileNetV3^33^. We then evaluate the performance of the segmentation models for bridge mask prediction ${\hat{Y}}'$ on the historical map $X'$. Specifically, we compare ${\hat{Y}}'$ with the groundtruth $Y'$ of the historical map that is manually annotated only for the evaluation purpose. Table 2 summarize the result, where the proposed framework achieved $F1_{inst:0.1} = 0.725$ and $F1_{inst:0.01} = 0.734$. This result demonstrates that geographic features in historical maps can indeed be learned effectively using only style-transferred maps and contemporary annotations in the proposed semi-supervised framework.

**Table 2:** Quantitative evaluation of the U-Net models trained on the synthetic historical maps. The synthesize historical maps are produced using style transfer models Pix2Pix, CycleGAN, and CUT, respectively. Results show that CUT outperforms the other two style-transfer models in generating synthetic historical maps for bridge segmentation use.

U-Net Trained on	$P_{px}$	$R_{px}$	$F1_{px}$	$IoU_{px}$	$P_{inst:0.1}$	$R_{inst:0.1}$	$F1_{inst:0.1}$	$F1_{inst:0.01}$	#Bridges
SynHM-Pix2Pix Training	0.053	0.004	0.008	0.004	0.054	0.003	0.005	0.008	56
SynHM-CycleGAN Training	**0.729**	0.248	0.371	0.227	0.863	0.315	0.461	0.485	422
SynHM-CUT Training	0.641	**0.468**	**0.541**	**0.371**	**0.873**	**0.619**	**0.725**	**0.743**	**818**

* SynHM stands for synthetic historical map.

Significant values are in [bold].

**Figure 8:**
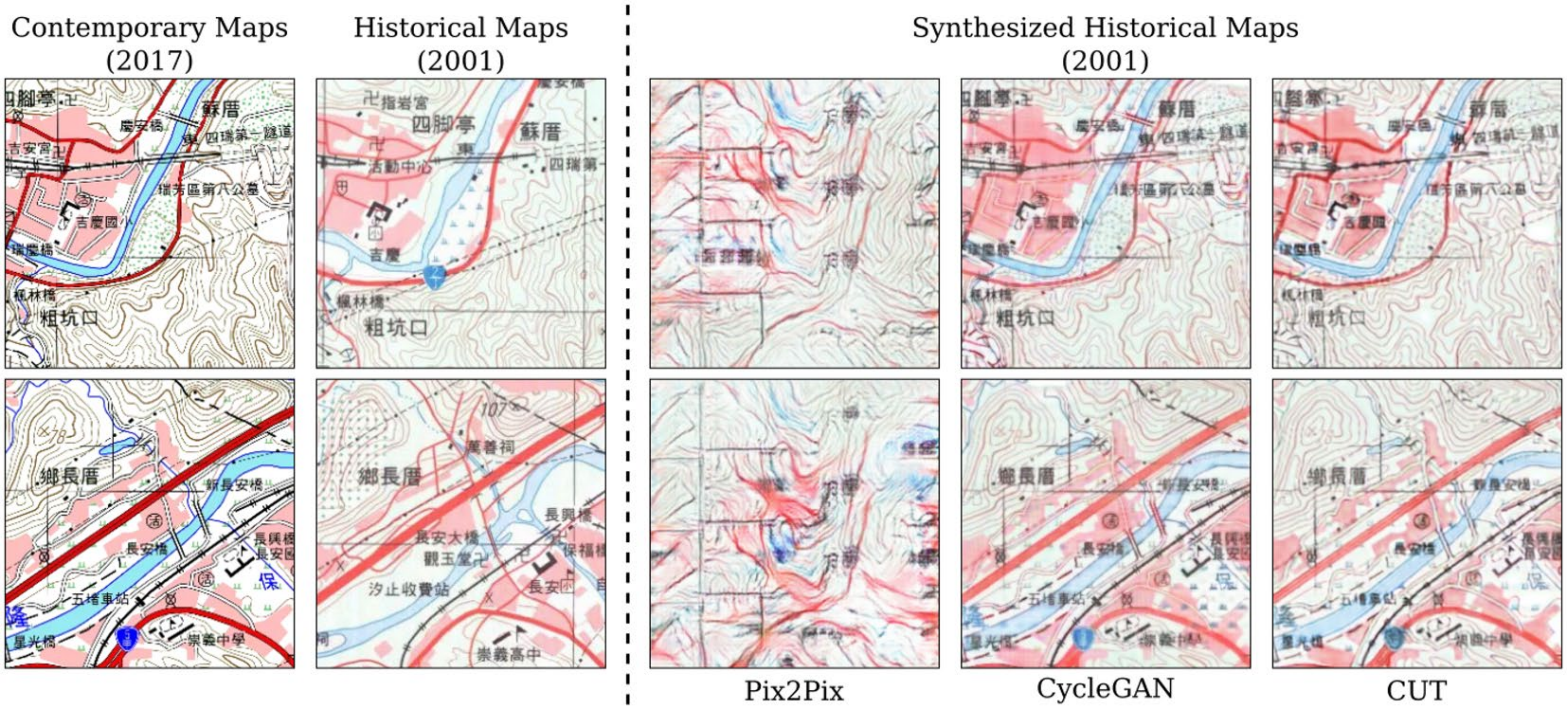
Examples of the synthesized historical maps generated by Pix2Pix^29^, CycleGAN^30^, and CUT^21^**.** Although the map tiles produced by Pix2Pix are obviously broken, CycleGAN and CUT transferred the contemporary map tiles to the historical style successfully. If we focus on the bridge symbols in the synthesized tiles, we can find that the tiles made by CUT are much more like the historical map than those made by CycleGAN. † The maps in this figure are provided by *Taiwan Historical Maps System* (https://gis.sinica.edu.tw/tileserver/) with permission.

*Comparison of the style transfer models used in Stage 1* We deployed three image-to-image translation models in the first stage of the framework to see the effectiveness of different models. Pix2Pix^29^, CycleGAN^30^, and CUT^21^ were selected to compare the performance between different transferring strategies. We trained the networks in the direction translating from the contemporary map *X* into the historical map $X'$ to produce synthesized historical map ${\hat{X}}'$. Figure 8 shows some synthesized historical map tiles. Clearly, the result of Pix2Pix did not work as well as other methods. The results of CycleGAN and CUT showed promising transferred style for synthesizing historical maps visually. To quantitatively evaluate performance, we trained U-Net models for each synthesized historical map from different translation models and tested the predictions of the U-Nets with the historical map. The quantitative evaluation is shown in Table 2, and it turns out that the performance of the model trained with CUT synthesized map outperformed the others. Although the U-Net trained with SynHM-CycleGAN with higher pixel-level precision $P_{px} = 0.729$, it failed to find sufficient target features, which is shown in $R_{px} = 0.248$ and $\#Bridges = 422$, that it lose to the one trained with SynHM-CUT on both pixel-level and instance-level F1-scores. In the training phase, it takes about 15.7 ms for Pix2Pix, 287.2 ms for CycleGAN, and 207.2 ms for CUT to train an image, respectively. For the synthesizing phase, it takes 11.7 ms for Pix2Pix, 37.95 ms for CycleGAN, and 15.2 ms for CUT to generate an image, respectively. In summary, the result indicates that CUT learns the relationship between the contemporary map and the historical map better than Pix2Pix and CycleGAN do, and learns that more efficient than CycleGAN.

*Comparison of the semantic segmentation models used in Stage 2* To determine which segmentation model is good at extracting geographic features in semi-supervised circumstances, we employed four semantic segmentation models, namely, U-Net^24^, fully-convolutional network (FCN)^31^, DeepLabV3^32^, and MobileNetV3^33^. We trained the segmentation models on CUT-synthesized historical map tiles and the contemporary masks. In the training phase, it takes about 14.3 ms for MobileNetV3, 40.6 ms for FCN, 53 ms for DeepLabV3, and 27 ms for U-Net to train an image, respectively. In the testing phase, it takes about 9 ms for MobileNetV3, 17.8 ms for FCN, 24.3 ms for DeepLabV3, and 13.2 ms for U-Net to predict an image, respectively. Table 3 shows the quantitative comparison result. These results show that all the segmentation models except MobileNetV3 make the prediction correctly with $P_{inst:0.1} \ge 0.75$. In particular, the U-Net produces better bridge segmentation predictions with $R_{inst:0.1} = 0.619$.

**Table 3:** Results from different semantic segmentation models. The U-Net outperforms other state-of-the-art segmentation models in this problem on every metrics. Since the dataset suffers from insufficient labels and imbalanced classification, the models with higher model complexity can easily overfit the transferred data.

Segmentation Model	$P_{px}$	$R_{px}$	$F1_{px}$	$IoU_{px}$	$P_{inst:0.1}$	$R_{inst:0.1}$	$F1_{inst:0.1}$	$F1_{inst:0.01}$	$\#Bridges$
MobileNetV3	0.479	0.135	0.211	0.118	0.608	0.149	0.239	0.304	283
FCN	0.617	0.361	0.455	0.295	0.813	0.405	0.541	0.589	578
DeepLabV3	0.625	0.323	0.426	0.270	0.786	0.380	0.513	0.578	560
U-Net	**0.641**	**0.468**	**0.541**	**0.371**	**0.873**	**0.619**	**0.725**	**0.743**	**818**

Significant values are in [bold].

### Evaluation of all combinations between style transfer models and semantic segmentation models

Extensive experiments are conducted for the comprehensive comparisons of 12 combinations among 4 segmentation models and 3 synthesized datasets for style transfer model training. To make the comparisons fair, we fix the training epoch to 400 for all runs, which ensures that the segmentation models perform learning on each dataset with the same visibility. In addition, we train each combination for 10 times with different random seed to obtain robust statistical results. Table 4 reports the $F1_{inst:0.1}$ scores for this comprehensive comparison. Additional results and details are available in in the supplementary file. One can clearly observe that segmentation models cannot learn well from the Pix2Pix synthesized dataset, which is reflected in the all zero outcomes in Table 4. The best scoring combination is still the U-Net trained on CUT synthesized dataset, whose $F1_{inst:0.1}$ score ($0.662 \pm 0.008$) is 57.3 % higher than the comparative score ($0.089 \pm 0.065$) of the least valid combination (the MobileNetV3 trained on CycgleGAN synthesized dataset).

**Table 4:** Comprehensive comparisons from the 12 combinations among the 4 segmentation models and 3 synthesized datasets for style transfer model training. The statistical $F1_{inst:0.1}$ are reported as the mean value of 10 independent runs with the standard deviation for each configuration.

Trained on Model	SynHM-Pix2Pix Training	SynHM-CycleGAN Training	SynHM-CUT Training
MobileNetV3	$0.000 \pm 0.000$	$0.089 \pm 0.065$	$0.143 \pm 0.094$
DeepLabV3	$0.000 \pm 0.000$	$0.175 \pm 0.092$	$0.459 \pm 0.185$
FCN	$0.000 \pm 0.000$	$0.341 \pm 0.034$	$0.567 \pm 0.026$
U-Net	$0.000 \pm 0.000$	$0.366 \pm 0.033$	**0.662** ± **0.008**

* SynHM stands for synthetic historical map.

Significant values are in [bold].

## Discussions

In this paper, we demonstrate that the proposed framework can learn target geographic features on historical maps without the need of annotations there. Results from Table 1 show that the training of supervised semantic segmentation models highly rely on labeled data, and without labeled data the models are unable to extract information across years. However, by fusing annotations with the transferred map tiles in the first stage, our two-stage framework can eliminate the dependence on labeled data for targeted year. The bridge segmentation model trained in our framework on a 2017 map can accurately locate bridges on the 2001 historical map without using annotations on the 2001 map. Finally, this framework can be extended to learn additional geographic features including roads, highways, land uses, etc. for geographical study on maps across years.

*Performance analysis* Table 4 shows that models trained on different synthetic dataset could have significant gaps no matter what segmentation model is used. For Pix2Pix style transfer, it is hard to obtain useful semantic information for training the segmentation models, as no specific constrains can be enforced on the model output. On the other hand, CUT leverages mutual information between source and destination datasets, which leads to the superior results in the experiment. We also observe that U-Net models are more stable, as the standard deviations of U-Net based combinations are much lower than the ones of other models. The downward and upward scaling constrains of U-Net can effectively regularize the semantic segmentation outcomes. In summary, refined constrains of the input synthetic dataset and the segmentation capability are the two key factors toward the success of the two-stage, semi-supervised, map style transfer analysis approach.

**Figure 9:**
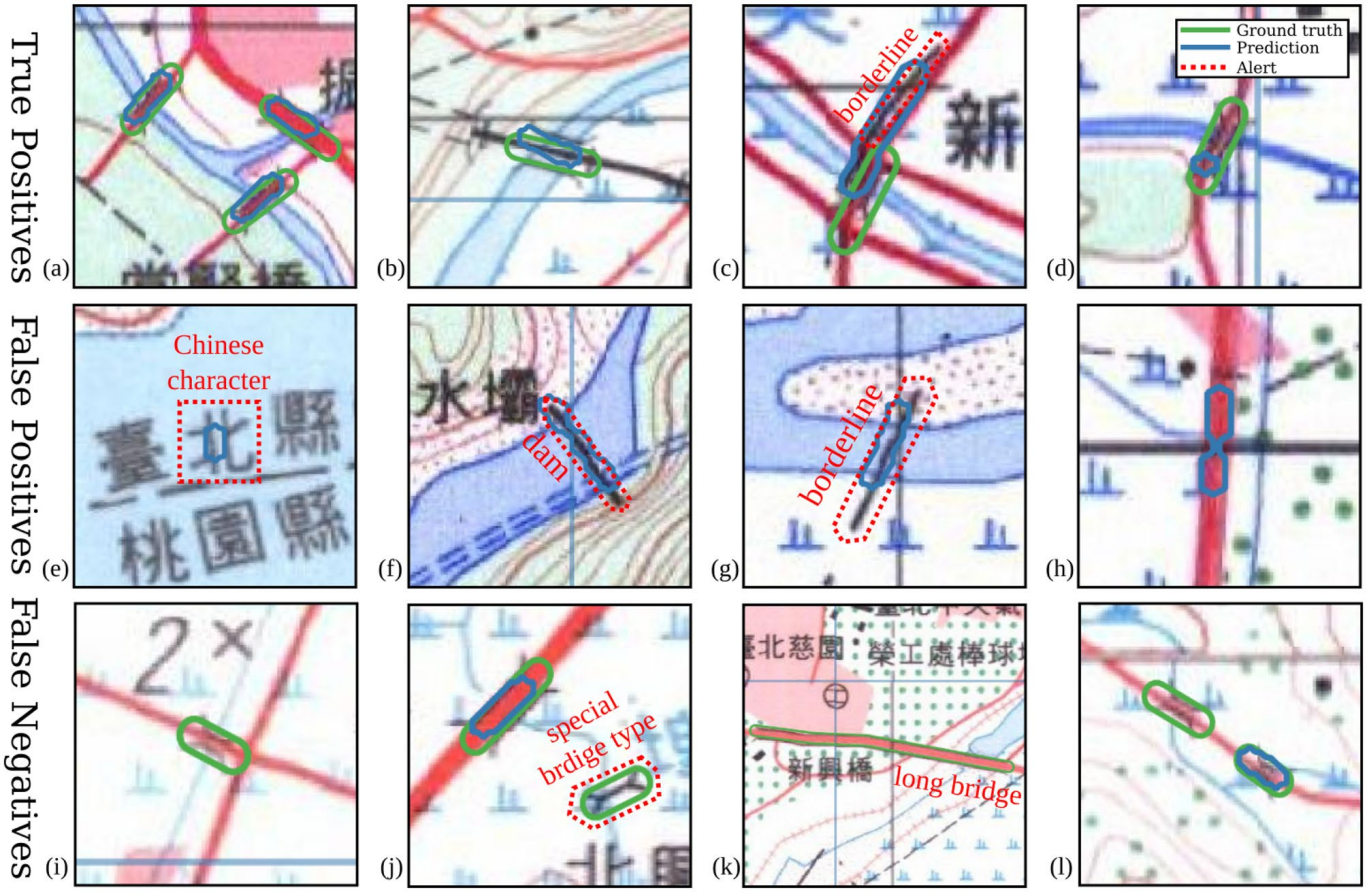
The examples from the results of the proposed framework. The contours in blue indicate the predicted bridges, and the ones in green represent the bridge groundtruth. (**a**–**d**) show the true positive examples. Although some predictions are influenced by other geographic features (such as borderlines in (**c**)) and some predict only heads of bridges in (**d**), most predictions intersect bridges successfully like the ones in (**a**) and (**b**). (**e**–**h**) show the false positive examples. Some geographic features are easily confused with bridges, e.g. a Chinese character on a river in (**e**), the dam in (**f**), and the borderline crossing a waterbody in (**g**). For some false-positive cases, such as in (**h**), the reasons for being recognized as bridges cannot be identified. (**i**–**l**) show the false negative examples. Relatively small and special bridges are hard to be detected, as shown (**i**), (**j**), and (**k**). In (**l**), there are two similar bridges but only one is recognized. † The maps in this figure are provided by *Taiwan Historical Maps System* (https://gis.sinica.edu.tw/tileserver/) with permission.

*Error analysis* We next discuss the failure cases of bridge segmentation and point out the source and characteristics of these errors. Results from Tables 2 and 3 suggest that the U-Net trained on SynHM-CUT is the best performing bridge segmentation model in the cross-year semi-supervised training experiments, so we focus the discussion on the failure cases of the U-Net. Figure 9 shows representative examples of the U-Net bridge segmentation trained on the transferred 2017 contemporary map and tested on the 2001 historical map. Most erroneous predictions are due to the ambiguous geographic features such as roads or highway segments that resemble bridges in the map. Bridge segmentation performance is also affected by the wrongly recognized background patterns, bridges that appear to be too short or too small, and the mixture of complicated representations of geographic features (such as highways cross bridges in the same intersection). The proposed style-transfer of maps can degrade the map quality and result in blurry maps with fixed features. In summary, the weaknesses are mostly due to the shape imbalance of target features and the low quality of detail of transferred map tiles.

*Limitations* of the proposed method are summarized in the following. First, the efficiency for learning geographic features across years is limited by the pairwise style transfer model. Since the style transfer network needs to be trained specifically for each pair of source and target map styles, only a pairwise mapping between a source style of map and a target style can be learned to perform at a time. Secondly, the effectiveness of understanding geographic features across years is temporally dependent. We selected contemporary maps for the map with annotations because the vector data for contemporary maps is more available than the ones for historical maps. As a result, the newer maps are much easier to be learn.

*Future work* of this study includes improving the segmentation model which discriminates between noisy geographic features and the target features. The model should be ensured that special cases (such as extremely long bridges in this study) are included in the dataset. Furthermore, we want to make the style transfer model in the framework to be more geographic feature awareness in the future; the style transfer model should learn the transformation for the features representing the same geographic meaning across years. We envision a better representation for geographic features extraction across multiple years can be learned jointly. We plan to expand our experiments on the 1992-1994, 1985-1989, and 1957-1969 maps.

## Conclusion

In this work, we formulate a two-stage deep learning approach to address the insufficient annotations of supervised learning when applying machine learning to historic map study. We leverage state-of-the-art image style transfer and semantic segmentation algorithms in studying geographic features using historical maps from 1957 to 2017 in Taiwan. The image styles are transferred among maps, such that topographic maps together with corresponding annotations can be aggregated across years. Image semantic segmentation networks are applied to detect and localize bridges in the maps. Our framework based on U-Net trained using the style-transferred CUT maps achieves F1-score $F1_{inst:0.1} = 0.725$ and $F1_{inst:0.01} = 0.743$ for bridge segmentation, which demonstrates a case of the feasibility of map information transfer across years.

We envision that our approach can be extended to other research based on geographic and historical map analysis. The source of our study and dataset will be released upon paper acceptance. Geographers can use the proposed framework as a tool to measure land use changes by extracting geographic features from historical maps across years. Furthermore, the organized dataset is a perfect setting for studies of style transfer and semi-supervised learning. The framework also supports automatic map generation research as an evaluation metric that validates the generated maps. In summary, this paper boosts the research for historical maps understanding.

## Supplementary Information

Supplementary Information.

## Data Availability

Map tiles used in this paper are collected from Web Map Tile Service (WMTS) of the Center for Geographic Information Science, Research Center for Humanities and Social Sciences at Academia Sinica. The vector data for geographic features of Taiwan are downloaded from the National Land Surveying and Mapping Center of Taiwan, and the masks for training and testing are generated from the vector data. Moreover, the datasets used in this work are published with the paper as *slippy map* structures, which can be downloaded from the supplementary files of the paper.

## References

[1] Can YS, Gerrits PJ, Kabadayi ME (2021). Automatic detection of road types from the third military mapping survey of Austria-Hungary historical map series with deep convolutional neural networks. IEEE Access.

[2] Maxwell AE (2020). Semantic segmentation deep learning for extracting surface mine extents from historic topographic maps. Remote Sens..

[3] Garcia-Molsosa A (2021). Potential of deep learning segmentation for the extraction of archaeological features from historical map series. Archaeol. Prospect..

[4] Chen Y-Y (2019). Reconstructing Taiwan’s land cover changes between 1904 and 2015 from historical maps and satellite images. Scientific Rep..

[5] Zhu X, Goldberg AB (2009). Introduction to semi-supervised learning. Synth. Lect. Artif. Intell. Mach. Learn..

[6] Kingma DP, Mohamed S, Jimenez Rezende D, Welling M (2014). Semi-supervised learning with deep generative models. Adv. Neural Inform. Process. Syst..

[7] Berthelot, D (2019). Mixmatch: A holistic approach to semi-supervised learning. Adv. Neural Inform. Process. Syst..

[8] Pan SJ, Yang Q (2009). A survey on transfer learning. IEEE Trans. Knowl. Data Eng..

[9] Weiss K, Khoshgoftaar TM, Wang D (2016). A survey of transfer learning. J. Big data.

[10] Tan, C. *et al.* A survey on deep transfer learning. In *International Conference on Artificial Neural Networks*, 270–279 (Springer, 2018).

[11] Zhuang F (2020). A comprehensive survey on transfer learning. Proc. IEEE.

[12] Kolesnikov, A., Zhai, X. & Beyer, L. Revisiting self-supervised visual representation learning. In *Proceedings of the IEEE International Conference on Computer Vision*, 1920–1929 (2019).

[13] Liu, X. *et al.* Self-supervised learning: Generative or contrastive. *IEEE Transactions on Knowledge and Data Engineering* (2021).

[14] Jing L, Tian Y (2020). Self-supervised visual feature learning with deep neural networks: A survey. IEEE Trans. Pattern Anal. Mach. Intell..

[15] Uhl, J. H., Leyk, S., Chiang, Y.-Y., Duan, W. & Knoblock, C. A. Extracting human settlement footprint from historical topographic map series using context-based machine learning. In *International Conference of Pattern Recognition Systems*, 1–6 (IET, 2017).

[16] Uhl JH, Leyk S, Chiang Y-Y, Duan W, Knoblock CA (2018). Spatialising uncertainty in image segmentation using weakly supervised convolutional neural networks: A case study from historical map processing. IET Image Proc..

[17] Uhl JH, Leyk S, Chiang Y-Y, Duan W, Knoblock CA (2019). Automated extraction of human settlement patterns from historical topographic map series using weakly supervised convolutional neural networks. IEEE Access.

[18] Duan, W., Chiang, Y.-Y., Knoblock, C. A., Leyk, S. & Uhl, J. H. Automatic generation of precisely delineated geographic features from georeferenced historical maps using deep learning. In *Proceedings of the AutoCarto* (2018).

[19] Duan W, Chiang Y-Y, Leyk S, Uhl JH, Knoblock CA (2020). Automatic alignment of contemporary vector data and georeferenced historical maps using reinforcement learning. Int. J. Geogr. Inf. Sci..

[20] Yau, N.-J. & Chuang, Y.-H. Analyzing taiwan bridge management system for decision making in bridge maintenance: A big data approach. In *International Joint Conference on Software Technologies*, vol. 1, 1–6 (IEEE, 2015).

[21] Park, T., Efros, A. A., Zhang, R. & Zhu, J.-Y. Contrastive learning for unpaired image-to-image translation. In *European Conference on Computer Vision*, 319–345 (Springer, 2020).

[22] Goodfellow I (2014). Generative adversarial nets. Adv. Neural Inform. Process. Syst..

[23] Oord, A. v. d., Li, Y. & Vinyals, O. Representation learning with contrastive predictive coding. *arXiv preprint* arXiv:1807.03748 (2018).

[24] Ronneberger, O., Fischer, P. & Brox, T. U-net: Convolutional networks for biomedical image segmentation. In *International Conference on Medical Image Computing and Computer-Assisted Intervention*, 234–241 (Springer, 2015).

[25] Berman, M., Triki, A. R. & Blaschko, M. B. The Lovász-softmax loss: A tractable surrogate for the optimization of the intersection-over-union measure in neural networks. In *Proceedings of the IEEE Conference on Computer Vision and Pattern Recognition*, 4413–4421 (2018).

[26] Kingma, D. P. & Ba, J. Adam: A method for stochastic optimization. *arXiv preprint* arXiv:1412.6980 (2014).

[27] Mapbox. Meet RoboSat. https://blog.mapbox.com/meet-robosat-af42530f163f (2018).

[28] QGIS Development Team. QGIS geographic information system. https://www.qgis.org (2021).

[29] Isola, P., Zhu, J.-Y., Zhou, T. & Efros, A. A. Image-to-image translation with conditional adversarial networks. In *Proceedings of the IEEE Conference on Computer Vision and Pattern Recognition*, 1125–1134 (2017).

[30] Zhu, J.-Y., Park, T., Isola, P. & Efros, A. A. Unpaired image-to-image translation using cycle-consistent adversarial networks. In *Proceedings of the IEEE International Conference on Computer Vision*, 2223–2232 (2017).

[31] Long, J., Shelhamer, E. & Darrell, T. Fully convolutional networks for semantic segmentation. In *Proceedings of the IEEE Conference on Computer Vision and Pattern Recognition*, 3431–3440 (2015). 10.1109/TPAMI.2016.2572683 27244717

[32] Chen, L.-C., Papandreou, G., Schroff, F. & Adam, H. Rethinking atrous convolution for semantic image segmentation. *arXiv preprint* arXiv:1706.05587 (2017).

[33] Howard, A. *et al.* Searching for mobilenetv3. In *Proceedings of the IEEE International Conference on Computer Vision*, 1314–1324 (2019).

