# Supplementary material for: Semi-supervised learning for topographic map analysis over time: a study of bridge segmentation
Source: Sci Rep. 2022 Nov 8;12:18997. doi: 10.1038/s41598-022-23364-w (PMC9643415; doi:10.1038/s41598-022-23364-w)
Supplement: Supplementary file 1 — Supplementary Information. [file 41598_2022_23364_MOESM1_ESM.pdf]

Semi-Supervised Learning for Topographic Map Analysis Over Time – A Study of Bridge Segmentation: Supplementary information

Cheng-Shih Wong[1], Hsiung-Ming Liao[1], Richard Tzong-Han Tsai[1,+], and Ming-Ching Chang[1,2,+]

[1]Center for Geographic Information Science, Research Center for Humanities and Social Sciences, Academia Sinica, Taipei, 115201, Taiwan
[2]Computer Science Department, University at Albany, State University of New York, Albany, NY 12222, USA
[+]Corresponding authors: thtsai@g.ncu.edu.tw, mchang2@albany.edu

1 Supplementary materials

Trained on	Tested on	Seg. Model	P_{px}	R_{px}	$F1_{px}$	IoU_{px}
CM Training	CM Testing	U-Net	0.845	0.764	0.803	0.670
	CM-Taichung	U-Net	0.986	0.373	0.541	0.371
	HM Testing	U-Net	0.849	0.003	0.007	0.003
SynHM-Pix2Pix Training	HM Testing	U-Net	0.053	0.004	0.008	0.004
SynHM-CycleGAN Training	HM Testing	U-Net	0.729	0.248	0.371	0.227
SynHM-CUT Training	HM Testing	U-Net	0.641	0.468	0.541	0.371
		FCN	0.617	0.361	0.455	0.295
		DeepLabV3	0.625	0.323	0.426	0.270
		MobileNetV3	0.479	0.135	0.211	0.118

* CM stands for contemporary map; HM stands for historical map; SynHM stands for synthesized historical map.

Table S1. Full pixel-level evaluation table for the experiments in the paper.

Trained on	Tested on	Seg. Model	$P_{inst:0.1}$	$R_{inst:0.1}$	$F1_{inst:0.1}$	$P_{inst:0.01}$	$R_{inst:0.01}$	$F1_{inst:0.01}$	#Bridges
CM Training	CM Testing	U-Net	0.935	0.820	0.874	0.965	0.832	0.893	960
	CM-Taichung	U-Net	0.966	0.898	0.931	0.981	0.906	0.942	1783
	HM Testing	U-Net	0.000	0.000	0.000	1.000	0.003	0.005	3
SynHM-Pix2Pix Training	HM Testing	U-Net	0.054	0.003	0.005	0.089	0.004	0.008	56
SynHM-CycleGAN Training	HM Testing	U-Net	0.863	0.315	0.461	0.910	0.330	0.485	422
SynHM-CUT Training	HM Testing	U-Net	0.873	0.619	0.725	0.900	0.633	0.743	818
		FCN	0.813	0.405	0.541	0.893	0.440	0.589	578
		DeepLabV3	0.786	0.380	0.513	0.895	0.427	0.578	560
		MobileNetV3	0.608	0.149	0.239	0.777	0.189	0.304	283

* CM stands for contemporary map; HM stands for historical map; SynHM stands for synthesized historical map.
#Bridges shows the number of recognized bridges.

Table S2. Full instance-level evaluation table for the experiments in the paper.

Model	Trained on	P_{px}	R_{px}	A_{px}	IoU_{px}	$F1_{px}$
MobileNetV3	SynHM-Pix2Pix Training	0.000 ± 0.000	0.000 ± 0.000	0.000 ± 0.000	0.000 ± 0.000	0.000 ± 0.000
	SynHM-CycleGAN Training	0.450 ± 0.170	0.056 ± 0.033	0.999 ± 0.000	0.052 ± 0.031	0.098 ± 0.054
	SynHM-CUT Training	0.534 ± 0.103	0.085 ± 0.050	0.999 ± 0.000	0.077 ± 0.043	0.141 ± 0.073
DeepLabV3	SynHM-Pix2Pix Training	0.000 ± 0.000	0.000 ± 0.000	0.000 ± 0.000	0.000 ± 0.000	0.000 ± 0.000
	SynHM-CycleGAN Training	0.689 ± 0.243	0.096 ± 0.049	0.899 ± 0.316	0.092 ± 0.046	0.166 ± 0.081
	SynHM-CUT Training	0.561 ± 0.200	0.293 ± 0.119	0.899 ± 0.316	0.242 ± 0.093	0.381 ± 0.142
FCN	SynHM-Pix2Pix Training	0.000 ± 0.000	0.000 ± 0.000	0.000 ± 0.000	0.000 ± 0.000	0.000 ± 0.000
	SynHM-CycleGAN Training	0.693 ± 0.031	0.190 ± 0.023	0.999 ± 0.000	0.175 ± 0.019	0.297 ± 0.027
	SynHM-CUT Training	0.591 ± 0.025	0.382 ± 0.036	0.999 ± 0.000	0.301 ± 0.020	0.463 ± 0.023
U-Net	SynHM-Pix2Pix Training	0.000 ± 0.000	0.000 ± 0.000	0.000 ± 0.000	0.000 ± 0.000	0.000 ± 0.000
	SynHM-CycleGAN Training	0.738 ± 0.031	0.181 ± 0.018	0.999 ± 0.000	0.170 ± 0.016	0.291 ± 0.024
	SynHM-CUT Training	0.607 ± 0.026	0.486 ± 0.016	0.999 ± 0.000	0.369 ± 0.006	0.539 ± 0.006

* SynHM stands for synthesized historical map.

Table S3. **Part 1 of full details for the comprehensive experiment in the paper.**

Model	Trained on	$P_{inst:0.1}$	$R_{inst:0.1}$	$F1_{inst:0.1}$	$P_{inst:0.01}$	$R_{inst:0.01}$
MobileNetV3	SynHM-Pix2Pix Training	0.000 ± 0.000	0.000 ± 0.000	0.000 ± 0.000	0.000 ± 0.000	0.000 ± 0.000
	SynHM-CycleGAN Training	0.462 ± 0.198	0.050 ± 0.039	0.089 ± 0.065	0.582 ± 0.221	0.062 ± 0.044
	SynHM-CUT Training	0.617 ± 0.125	0.085 ± 0.061	0.143 ± 0.094	0.752 ± 0.131	0.101 ± 0.069
DeepLabV3	SynHM-Pix2Pix Training	0.000 ± 0.000	0.000 ± 0.000	0.000 ± 0.000	0.000 ± 0.000	0.000 ± 0.000
	SynHM-CycleGAN Training	0.705 ± 0.250	0.102 ± 0.056	0.175 ± 0.092	0.800 ± 0.282	0.116 ± 0.063
	SynHM-CUT Training	0.661 ± 0.233	0.360 ± 0.161	0.459 ± 0.185	0.720 ± 0.253	0.386 ± 0.169
FCN	SynHM-Pix2Pix Training	0.000 ± 0.000	0.000 ± 0.000	0.000 ± 0.000	0.000 ± 0.000	0.000 ± 0.000
	SynHM-CycleGAN Training	0.786 ± 0.028	0.219 ± 0.029	0.341 ± 0.034	0.850 ± 0.032	0.235 ± 0.031
	SynHM-CUT Training	0.732 ± 0.027	0.464 ± 0.042	0.567 ± 0.026	0.790 ± 0.028	0.497 ± 0.046
U-Net	SynHM-Pix2Pix Training	0.000 ± 0.000	0.000 ± 0.000	0.000 ± 0.000	0.000 ± 0.000	0.000 ± 0.000
	SynHM-CycleGAN Training	0.794 ± 0.030	0.238 ± 0.026	0.366 ± 0.033	0.821 ± 0.031	0.246 ± 0.025
	SynHM-CUT Training	0.718 ± 0.024	0.615 ± 0.018	0.662 ± 0.008	0.743 ± 0.025	0.641 ± 0.019

* SynHM stands for synthesized historical map.

Table S4. **Part 2 of full details for the comprehensive experiment in the paper.**

Model	Trained on	$F1_{inst:0.01}$	Bridges
MobileNetV3	SynHM-Pix2Pix Training	0.000 ± 0.000	0.000 ± 0.000
	SynHM-CycleGAN Training	0.110 ± 0.073	106.444 ± 47.022
	SynHM-CUT Training	0.170 ± 0.106	146.500 ± 99.041
DeepLabV3	SynHM-Pix2Pix Training	0.000 ± 0.000	0.000 ± 0.000
	SynHM-CycleGAN Training	0.199 ± 0.104	139.900 ± 76.237
	SynHM-CUT Training	0.495 ± 0.196	523.700 ± 234.427
FCN	SynHM-Pix2Pix Training	0.000 ± 0.000	0.000 ± 0.000
	SynHM-CycleGAN Training	0.367 ± 0.037	299.222 ± 49.547
	SynHM-CUT Training	0.608 ± 0.030	682.400 ± 84.782
U-Net	SynHM-Pix2Pix Training	0.000 ± 0.000	0.000 ± 0.000
	SynHM-CycleGAN Training	0.378 ± 0.031	319.778 ± 35.131
	SynHM-CUT Training	0.688 ± 0.008	913.600 ± 50.923

* SynHM stands for synthesized historical map.
#Bridges shows the number of recognized bridges.

Table S5. Part 3 of full details for the comprehensive experiment in the paper.

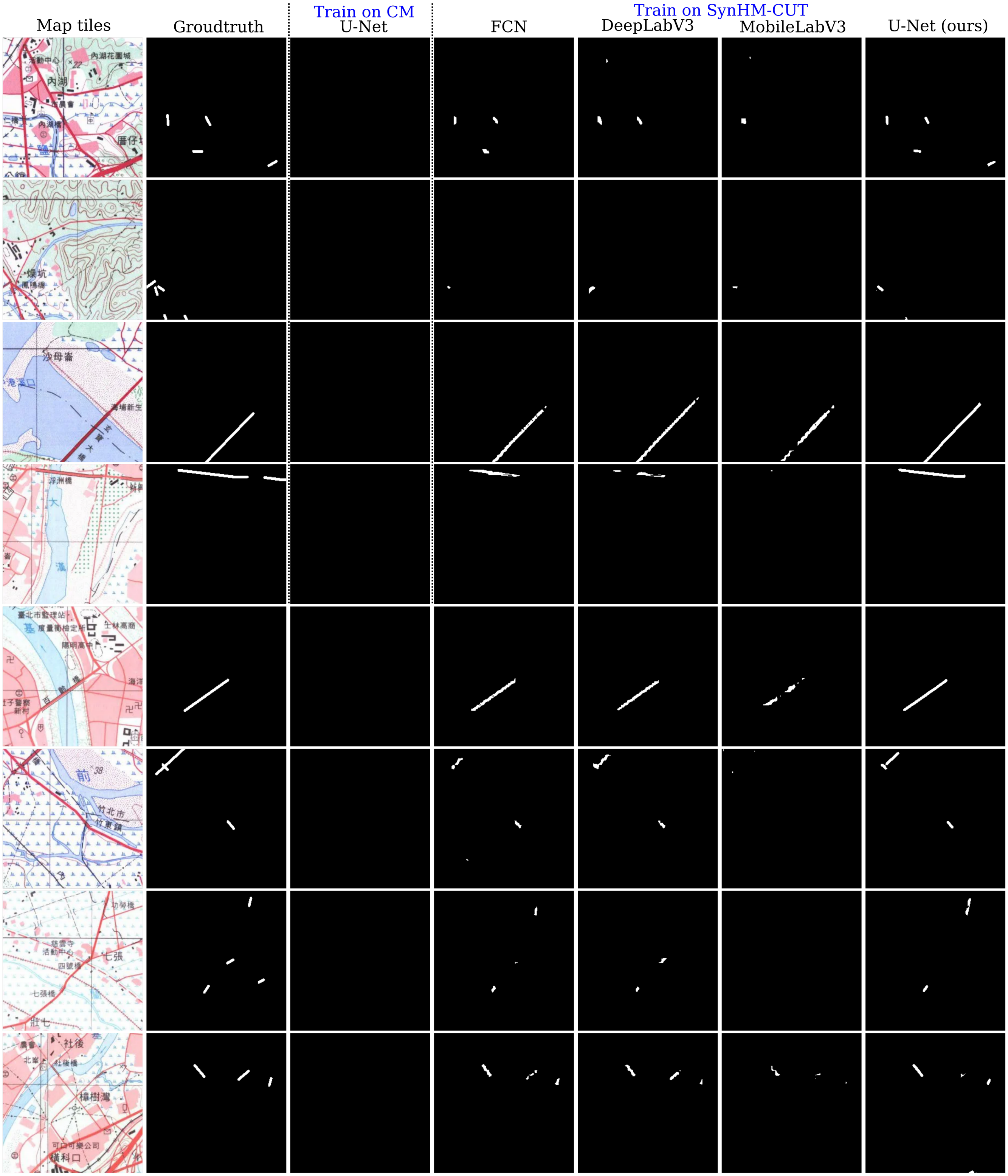

* CM stands for contemporary map; SynHM-CUT stands for CUT synthesized historical map.

Figure S1. Examples of results of semantic segmentation models are selected randomly. † The maps in this figure are provided by *Taiwan Historical Maps System* (`https://gis.sinica.edu.tw/tileserver/`) with permission.

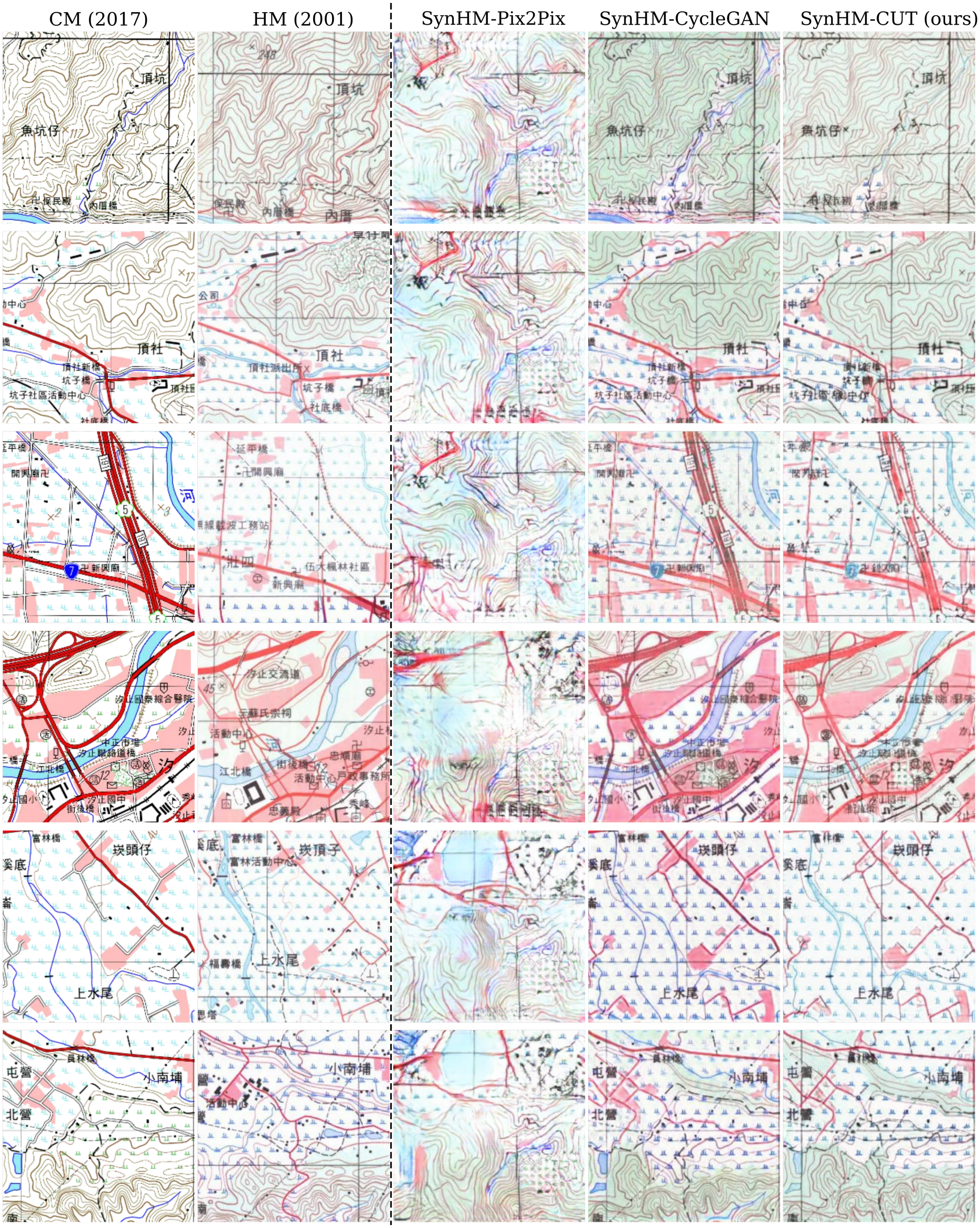

* CM stands for contemporary map; HM stands for historical map; SynHM stands for synthesized historical map.

Figure S2. Examples of results of style transfer models are selected randomly. † The maps in this figure are provided by *Taiwan Historical Maps System* (`https://gis.sinica.edu.tw/tileserver/`) with permission.
